# Malformations congénitales à Lubumbashi (République Démocratique du Congo): à propos de 72 cas observés et plaidoyer en faveur du développement d'un Registre National des Malformations Congénitales et d'un Centre National de Référence de Génétique Humaine

**Published:** 2012-12-19

**Authors:** Toni Kasole Lubala, Mick Yapongombo Shongo, Arthur Ndundula Munkana, Augustin Mulangu Mutombo, Sébastien Musanzayi Mbuyi, Félix Kitenge wa Momat

**Affiliations:** 1 Département de Pédiatrie, BP 1825, Faculté de Médecine, Université de Lubumbashi, République Démocratique du Congo; 2 Département de Gynécologie-Obstétrique, BP 1825, Faculté de Médecine, Université de Lubumbashi, République Démocratique du Congo; 3 Département de Chirurgie, BP 1825, Faculté de Médecine, Université de Lubumbashi, République Démocratique du Congo

**Keywords:** Malformations congénitales, génétique humaine, surveillance, prévention, Congenital malformations, human genetic, surveillance, prevention

## Abstract

En République Démocratique du Congo, les malformations congénitales constituent un véritable problème de santé publique. En effet, elles relancent le débat sur les effets de l'intensification de l'activité minière sur la santé de la reproduction. De 2009 à 2010, nous avons calculé une prévalence de 5.84 pour 1000 naissances. Les malformations du système nerveux central étaient les plus fréquentes (2.029 pour 1000) suivies des malformations des membres (1.055 pour 1000), et des fentes oro-faciales (0.811 pour 1000). Ces données sont certainement largement sous-estimées et les causes y relatives en République Démocratique du Congo ne sont ni surveillées, ni prévenues dans le cadre d'une politique gouvernementale. La mise en place d'un registre national et d'un centre national de génétique humaine de référence pourrait constituer un cadre rigoureux, organisé et structuré de surveillance et de prévention des malformations congénitales.

## Aux editeurs du Journal Panafricain de Médecine

L'accouchement d'un enfant malformé est vécu dans les sociétés Africaines comme un véritable drame compte tenu des considérations mystico-religieuses qui l'entourent d'une part et du véritable poids qu'elle constitue pour les familles d'autre part.

En République Démocratique du Congo, l'absence de structure organisée de diagnostic génétique, d'enregistrement et de prévention des cas en font un véritable défi de santé publique. De plus, elles posent avec acuité le problème des effets sur la santé de la reproduction de l'intensification de l'activité minière observée à Lubumbashi et ses environs et dans le reste de la province du Katanga. En effet, il existe de fortes présomptions quant aux effets tératogènes des minerais exploités dans cette province du pays. Ces présomptions sont liées aux taux anormalement élevés de ces minerais (Arsenic, Cadmium, Cobalt, Cuivre, Plomb et Uranium) retrouvés dans les urines de la population générale vivant à moins de 3-10 km des zones d'exploitation au Katanga [1].

En 2009, la ville de Lubumbashi a enregistré 55260 naissances vivantes [2]. Vingt-deux pour cent d'entre elles (12320) ont été surveillées dans 11 maternités sur les 80 que comptait la ville au moment de l’étude. Nous avons observé 72 cas sur 12320 naissances vivantes, soit une prévalence de 5.8 pour 1000. Notons que seules les malformations cliniquement visibles à la naissance ont été considérées. Les cardiopathies congénitales, qui représentent pourtant 40% de l'ensemble des malformations congénitales dans le monde n'ont pas fait partie de notre série. Ces chiffres sont donc certainement sous-estimés.

Dans un tout autre contexte (Belgique), une prévalence nettement supérieure à celle de notre série (25 pour 1000 naissances) a été rapportée [3]. Ceci s'explique très certainement par la création du registre des malformations congénitales du Hainaut et Namur affilié à "l'European Registry of Congenital anomalies and twins" (Eurocat). Ce registre, mis en place en Europe dans les années 60, à la suite du grand scandale de la phocomélie iatrogène induite par la thalidomide, a permis le recrutement plus rigoureux et plus exhaustif des cas, ainsi qu'une surveillance plus vigilante des nouveaux agents tératogènes.

Dans notre étude, les malformations du système nerveux étaient les plus fréquentes avec 2.029 pour 1000 naissances, suivies des malformations des membres avec 1.055 pour 1000 naissances et des fentes oro-faciales avec 0.8 pour 1000 naissances. Dans ces groupes, les spina-bifida, les fentes labiales et palatines ainsi que les pieds bots sont les plus représentées avec respectivement 0.568; 0.649 et 0.568 pour 1000 naissances (Tableau 1).

**Tableau 1:** La fréquence des malformations congénitales selon le système (classification ICD 10)

System	ICD code	N	Frequency/10000
**Nervous system**			
Neural Tube Defects:			
Anencephalus and similar	Q00	1	0.8
Encephalocele	Q01	1	0.8
Spina Bifida	Q05	7	5.68
Hydrocephaly	Q03	5	4.05
Microcephaly	Q02	5	4.05
Arhinencephaly / holoprosencephaly	Q04.1/Q04.2	6	4.87
Total		**25**	
**Eye**			
Anophthalmos	Q11.0	1	0.8
Microphtalmos	Q11.1	1	0.8
Total		**2**	
**Ear**			
Anotia	Q16.0	1	0.8
Low set ears		6	4.87
Total		**7**	
**Respiratory**			
Choanal atresia	Q30.0	1	0.8
**Orofacial clefts**			
Cleft lip with /without cleft palate	Q36/Q37	8	6.49
Cleft palate	Q35	2	1.6
Total		**10**	
**Digestive system**			
Ano-rectal atresia and stenosis	Q42	4	3.24
Total		**4**	
**Abdominal wall defects**			
Gastroschisis	Q79.3	2	1.6
Omphalocele	Q79.2	3	2.43
Total		**5**	
**Genital**			
Hypospadias	Q54	2	1.6
Indeterminate sex	Q56	3	2.43
Total		**5**	
**Limb**			
**Lower limb reduction**	**Q72**		
Absence of both lower leg and foot	Q72.2	1	0.8
Club foot - talipes equinovarus	Q66.0	7	5.68
Polydactyly	Q69	5	4.05
Total		**13**	
**TOTAL**		**72**	

Ces malformations congénitales, connues pour être d'origine multifactorielle [4, 5], peuvent dans certains cas, être prévenues par des mesures simples et accessibles même aux pays à ressources limitées comme la République Démocratique du Congo. Parmi ces mesures, citons la sensibilisation contre la consommation d'alcool durant la grossesse ainsi que la supplémentation périconceptionnelle en acide folique [6, 7].

S'agissant de cette dernière stratégie, l'expérience Irlandaise a permis d'obtenir une réduction de la prévalence des malformations du tube neural de 4.69/1000 naissances à 1.16/1000 entre 1980 et 1994 grâce à la prise effective d'acide folique en période péri-conceptionnelle. Des résultats semblables ont été observés aux Etats-Unis et en Chine [8–10]. La détermination de l'efficacité des protocoles de supplémentation a été rendue possible grâce à l'existence de registres de malformations organisés, structurés et rigoureux.

S'inspirant de l'expérience européenne, le registre des malformations congénitales en République Démocratique du Congo aurait donc pour but de fournir une information épidémiologique sur les malformations congénitales apparaissant dans le pays, de faciliter la détection précoce de nouveaux agents tératogènes, d’évaluer l'efficacité des programmes de prévention primaire, de servir de référence aux professionnels de la santé et de participer à la recherche sur les causes des malformations congénitales et sur leur prévention et leur traitement.

Par ailleurs, l'absence de service de Génétique humaine en République Démocratique du Congo rend quasi impossible le conseil génétique. La création d'un Centre de Génétique humaine de référence aux côtés du registre national des malformations congénitales constitue donc, de notre point de vue, un impératif de santé publique. Ce centre pourrait être organisé grâce à l'apport multidisciplinaire de Pédiatres, Généticiens, dysmorphologistes, foetopathologistes, Obstétriciens et chirurgiens nationaux et internationaux. Sa création devrait s'inscrire dans le cadre d'une politique gouvernementale de surveillance, de prévention et de traitement des malformations congénitales.

## Conclusion

La surveillance, la prévention ainsi que la prise en charge des malformations congénitales posent un véritable challenge en République Démocratique du Congo, société pronataliste où il n'existe ni registre des malformations congénitales, ni Centre de Génétique humaine de référence. Nous pensons qu'il est d'autant plus vital d'organiser de telles structures qu'il existe de fortes présomptions quant aux effets tératogènes des minerais exploités dans ce pays.

## References

[1] Banza CL, Nawrot TS, Haufroid V, Decrée S (2009). High human exposure to cobalt and other metals in Katanga, a mining area of the Démocratic Républic of Congo. Environmental research..

[2] (2009). Ministère de la Santé publique, Rapport SNIS.

[3] Gillerot Y, Mols M (2009). Quinze années de surveillance des malformations congénitales dans le Hainaut et dans la province de Namur: Enseignements et recommandations. Services publics de Wallonie.

[4] Mossey PA, Little J, Munger RG, Dixon MJ, Shaw WC (2009). Cleft lip and palate. Lancet..

[5] Wyszynski DF (2006). Neural tube defects: from origin to treatment..

[6] O'Leary CM, Nassar N, Kurinczuk JJ (2010). Prenatal Alcohol Exposure and Risk of Birth Defects. Pediatrics..

[7] De Wals P, Tairou F, Van Allen MI, Soo-Hong Uh (2007). Reduction in Neural-Tube Defects after acid folic fortification. N Engl J Med.

[8] Thame G, Guerra-Shinohara EM, Moron AF (2002). Serum Folate by Two Methods in Pregnant Women Carrying Fetuses with Neural Tube Defects. Clinical Chemistry..

[9] Margaret A Honein, Leonard J (2001). Impact of Folic Acid Fortification of the US Food Supply on the Occurrence of Neural Tube Defects. JAMA..

[10] Robert J Berry, Zhu Li, Daviderickson J, Song Li, Cynthia A (1999). Prevention of neural-tube defects with folic acid in china. N Engl J Med.

